# A Random Parameters Ordered Probit Analysis of Injury Severity in Truck Involved Rear-End Collisions

**DOI:** 10.3390/ijerph17020395

**Published:** 2020-01-07

**Authors:** Xiaojun Shao, Xiaoxiang Ma, Feng Chen, Mingtao Song, Xiaodong Pan, Kesi You

**Affiliations:** 1 The Key Laboratory of Road and Traffic Engineering, Ministry of Education Tongji University, Shanghai 201804, China; invincible_sxj@tongji.edu.cn (X.S.); fengchen@tongji.edu.cn (F.C.); 1710908@tongji.edu.cn (M.S.); 03013@tongji.edu.cn (X.P.); 2 Shanghai Municipal Engineering Design Institute (Group) Co., Ltd., Shanghai 200092, China; youkesi@smedi.com

**Keywords:** injury severity, truck-involved rear-end collision, random parameter ordered probit

## Abstract

Social and economic burdens caused by truck-involved rear-end collisions are of great concern to public health and the environment. However, few efforts focused on identifying the difference of impacting factors on injury severity between car-strike-truck and truck-strike-car in rear-end collisions. In light of the above, this study focuses on illustrating the impact of variables associated with injury severity in truck-related rear-end crashes. To this end, truck involved rear-end crashes between 2006 and 2015 in the U.S. were obtained. Three random parameters ordered probit models were developed: two separate models for the car-strike-truck crashes and the truck-strike-car crashes, respectively, and one for the combined dataset. The likelihood ratio test was conducted to evaluate the significance of the difference between the models. The results show that there is a significant difference between car-strike-truck and truck-strike-car crashes in terms of contributing factors towards injury severity. In addition, indicators reflecting male, truck, starting or stopped in the road before a crash, and other vehicles stopped in lane show a mixed impact on injury severity. Corresponding implications were discussed according to the findings to reduce the possibility of severe injury in truck-involved rear-end collisions.

## 1. Introduction

As reported by the World Health Organization, road traffic injuries are the 8th leading cause of death, leading to nearly 1.35 million deaths each year, causing great public health issues and environmental concerns [1]. As the dominant mode in short-distance shipments, according to the U.S. Department of Transportation [2], truck transport contributes almost 60% of the total weight of shipments in 2015, making it the largest share of freight transportation. However, owing to the unique attributes of trucks in terms of weight, height, length, and braking performance, large truck-involved crashes have drawn considerable attention to the government and the public in recent years. According to the National Highway Traffic Safety Administration [3], there is a 12% increase in the total number of people killed in large truck crashes over ten years from 2007 to 2017 in the United States.

Among these large truck-involved fatal crashes, 72% in 2017 are occupants of other vehicles, including passenger cars [3]. The consequences are often severe, not only for the physical harm but also for the costs, including property damage and social influence (such as travel delays). Hence, to prevent injuries and costs caused by large trucks, an examination of the hidden dangers and contributing factors to truck-passenger car crashes is required.

Past studies have examined occupant injuries of truck-involved rear-end crashes by considering the two categories, passenger cars strike trucks (P2T) and passenger cars stuck by trucks (T2P), jointly [4]. Although they share similar crash characteristics such as vulnerable passenger car occupants and unsafe driver actions, injury severities may vary according to different collision orders [5]. Moreover, using a combined dataset might omit important differences in contributing factors between P2T and T2P crashes.

With this in mind, this study focus on the relationships between contributing factors and occupant injury severity in the large truck-involved rear-end collisions through a comparative investigation based on the dataset from the National Automotive Sampling System (NASS) General Estimates Data System (GES) of the United States. The rear-end collisions are among the most frequent types of truck-passenger car crashes [2]. In addition, this type of collision has a higher injury severity rate as compared to non-truck-involved crashes [6].

This study adopted a random-parameters ordered probit approach to provide a deep understanding of the influence of contributing factors on occupant injury. This model considers the ordinal nature of injury data, and it is statistically superior to the fixed parameters ordered probit model as it accounts for possible unobserved factors [7,8,9]. The findings of this study will shed light on identifying the potential difference of contributing factors between truck as leading vehicle and passenger-car as the leading vehicle in rear-end collisions, therefore guide making tailored countermeasures to alleviate the resulting injury severity levels.

## 2. Literature Review

Previous studies on truck-involved crashes seek to understand the crash characteristics and injury severity under the impact of human behavior, vehicle performance, geometric roadway, and surroundings. With distinct objectives and databases, studies vary in different ways. Table 1 provides a summary of these studies in terms of the truck definition, dependent variable and scale, model, and key findings.

Three general observations can be made from Table 1. First, although injury severity from the police report was usually treated as the measure of crash outcome, some studies focused on drivers’ injuries [5,9,10,11,12,13,14] while others focused on the most severely injured occupant [15,16,17,18,19,20,21]. Second, from a methodological perspective, most studies accounted for the ordinal nature of the injury severity levels by using ordered discrete choice models [4,15,16,17,18], while others adopted unordered discrete choice models and machine learning methods such as classification and regression trees [6,10,11,12,14,19,21,22]. Although the classic KABCO scale (K, A, B, C, and O refers to fatal, incapacitating, non-incapacitating, no visible injury but complain of pain, and no injury, respectively) is the optimal way for analysis, due to data limitations, the grouping of some adjacent levels is necessary to obtain a sufficient number of observations, usually resulting in a range of two to four levels [5,10,11,13,15,16,17,18]. Last, various truck definitions were adopted according to the characteristics of the accessed database, with a majority of studies using Gross Vehicle Weight Rating (GVWR) over 10,000 lb (equals to 10,000 pounds/44.5KN) as a measuring standard [5,10,11,12,15,17].

In general, some studies were trying to identify the significant influencing factors on injury severity in terms of driver features, vehicle, roadway, environmental conditions, and collision characteristics [10,11,12,17], while others focused on a specific aspect only such as weather and lighting condition [13,22]. There are also some studies focused on a specific collision type such as rear-end and single-vehicle crash [4,9,16], while others are concentrating on finding the injury severity difference between various locations [10,23]. In addition, crash frequency and safety evaluating approach related to larger trucks are also been examined [24,25]. Overall, these studies have contributed greatly to the understanding of contributing factors towards injury severity in truck-involved crashes.

However, few efforts were made to identify the influence of collision order, and the differences in injury severity between P2T and T2P rear-end crashes were largely overlooked. In light of the above discussion, this study assumes that there are unique contributing factors between truck as a leading vehicle and passenger car as a leading vehicle in rear-end crashes, as the similar results illustrated in other comparison studies [10,11,26,27]. Specifically, the objective of this study is twofold: (i) to investigate the differences of effects of factors that contribute to injury severity in truck-involved rear-end collisions, and (ii) to compare the model performance between combined dataset (model truck-involved rear-end crashes as a whole) and separate dataset (model P2T crashes and T2P crashes separately).

## 3. Data Description

Ten years of truck-involved crash records (2006–2015) were collected from NASS-GES, a nationally representative sample of police-reported crashes [28]. The NASS-GES data were randomly sampled and weighted from 60 geographic sites across the United States. Although a majority of crashes involving minor property damage only and no significant personal injury are not reported, the NASS-GES still works as a great database for analyzing highway safety problems.

A total of 10,455 records were obtained from the 10-year truck-involved crash database to investigate the potential contributing factors to injury severity. Each record represents the most severely injured occupant in the crash. Note that all observations were reserved if a crash resulted in several most severely injured levels. After screening out the records with incomplete information, we got a final dataset consisting of 8506 observations, which includes 4866 P2T records and 3640 T2P records.

Trucks in this study were defined as vehicles with Gross Vehicle Weight Rating (GVWR) greater than 10,000 lb. The dependent variable, injury severity, was described using the KABCO scale in the raw data set, which was regrouped into four levels to ensure each category had a decent number of observations. The descending order of injury level was considered to reduce bias and variability of the estimated parameters for the ordered probit model [29]. The scale is presented as follows:

- L3: Severe Injury (K&A: fatal and incapacitating injury);
- L2: Evident Injury (B: non-incapacitating injury);
- L1: Possible Injury (C: possible injury);
- L0: No Injury (O: property damage only).

Among 4866 injury records in the P2T crash, 4.7% occupants had a severe injury, 10.4% had an evident injury, 13.4% had a possible injury, and 71.5% had no injury. Among 3640 maximum injury records in the T2P crash, 3.7% occupants had a severe injury, 12.7% had an evident injury, 30.4% had a possible injury, and 53.2% had no injury or property damage only.

To test for possible collinearity, the authors conducted Pearson’s correlation test. Two pairs were found to be highly correlated, with a correlation parameter of 0.93 between the person type and seat position, and 0.69 between road surface condition and weather. Both correlations are self-explanatory since the driver can only sit in driver’s seat, and road surface status changes according to the weather condition (e.g., wet surface in rainy day and icy surface on a snowy day). Both the seat position and weather were discarded to avoid collinearity. In consequence, a total number of 36 variables were to be tested in models for this study. The variables were categorized into five groups: person characteristics, vehicle characteristics, roadway and environment, crash mechanism, and temporal characteristics. A summary of the statistics is presented in Table 2.

Note that all variables were in dummy coding (with values of 0 and 1). Therefore, the mean value can be interpreted as proportion. For example, the mean value for male variables in the P2T data set represents there are 69.9% of samples are male. These variables were subsequently examined in the injury severity model specifications of truck-involved rear-end collisions.

## 4. Methodology

This study focuses on identifying the differences of contributing factors to injury severity between P2T and T2P collisions. Four crash injury severity outcomes were considered: severe injury, evident injury, possible injury, and no injury. As two categories of the most frequently used approaches, both the ordered model and the unordered model have been widely applied to examine the impact of contributing factors on injury severity [4,10,12,14]. Given that both the ordered and unordered models have their strength as well as limitations, the current study uses the ordered probit model as it accounts for the indexed nature of injury severity levels [30].

The ordered probit model is derived by introducing a latent variable $y^{*}$ as a basis for modeling the injury severity of each observation, which can be defined as follows [31]:

(1) $$y^{*}=\beta^{\prime }X+\varepsilon$$

where X is a vector of independent variables considered, $\beta^{\prime }$ is a vector of estimable parameters, and ε is a random error term assumed to be normally distributed across observations with a mean equal to 0 and a variance equal to 1.

Given Equation (1), the dependent variable *y* is defined by the unobserved variable $y^{*}$ as follows:

(2) $$y=\{\begin{array}{l} 3\;if\;y^{*}\geq \mu_{2}\;\left(severe\;injury\right)\\ 2\;if\;\mu_{1}<y^{*}\leq \mu_{2}\;\left(evident\;injury\right)\\ 1\;if\;\mu_{0}<y^{*}\leq \mu_{1}\;\left(possible\;injury\right)\\ 0\;if\;y^{*}\leq \mu_{0}\;\left(no\;injury\right) \end{array}$$

where $\mu_{0}$ = 0, $\mu_{1}$, and $\mu_{2}$ are thresholds that are jointly estimated with $\beta^{\prime }$ parameters. Then the probability of each injury category for given variables can be described on the distribution of random error ε:

(3) $$\begin{array}{l} P\left(y=3\right)=1-\Phi \left(\mu_{2}-\beta^{\prime }X\right)\\ P\left(y=2\right)=\Phi \left(\mu_{2}-\beta^{\prime }X\right)-\Phi \left(\mu_{1}-\beta^{\prime }X\right)\\ P\left(y=1\right)=\Phi \left(\mu_{1}-\beta^{\prime }X\right)-\Phi \left(\mu_{0}-\beta^{\prime }X\right)\\ P\left(y=0\right)=\Phi \left(\mu_{0}-\beta^{\prime }X\right) \end{array}$$

However, this standard probit model might lead to potential bias by treating the parameters $\beta^{\prime }$ as a constant value across observations, which restricts each variable to have the same impact on every individual observation [32,33,34]. Therefore, to account for these circumstances, a random parameter ordered probit model was developed to capture the unobserved heterogeneity, which is achieved by adding a randomly distributed error term φ (e.g., a normally distributed term with mean = 0 and variance = $\sigma^{2}$) [35]:

(4) $$\beta^{*}=\beta +\varphi$$

Since the interpretation of the estimated coefficient β on injury severity is not straightforward, marginal effects were computed to measure the effect of one unit change in an independent variable on the probability of injury severity. This is usually used to measure the influence of a variable in an injury severity level while keeping all other variables constant. The marginal effects are calculated as follows [31]:

(5) $$\begin{array}{l} \frac{\partial P\left(y=3\right)}{\partial X}=\Phi (\mu_{2}-\beta^{\prime }X)\beta \\ \frac{\partial P\left(y=2\right)}{\partial X}=[\Phi \left(\mu_{1}-\beta^{\prime }X\right)-\Phi \left(\mu_{2}-\beta^{\prime }X\right)]\beta \\ \frac{\partial P\left(y=1\right)}{\partial X}=[\Phi \left(\mu_{0}-\beta^{\prime }X\right)-\Phi \left(\mu_{1}-\beta^{\prime }X\right)]\beta \\ \frac{\partial P\left(y=0\right)}{\partial X}=\Phi (\mu_{0}-\beta^{\prime }X)\beta \end{array}$$

## 5. Model Evaluation

To determine whether the separate models of P2T and T2P can be warranted, the likelihood ratio test is adopted [31]. The likelihood ratio statistics is defined as follows [36]:

(6) $${Χ}^{2}=-2\left[LL\left(\beta_{ALL}\right)-LL\left(\beta_{P2T}\right)-LL\left(\beta_{T2P}\right)\right]$$

where $LL\left(\beta_{ALL}\right)$, $LL\left(\beta_{P2T}\right)$, and $LL\left(\beta_{T2P}\right)$ are the log-likelihoods at the convergence of the joint data model, car-strike-truck model, and truck-strike-car model, respectively. The ${Χ}^{2}$ statistic is a $\chi^{2}$ distribution with the degrees of freedom d equal to the sum of the number of parameters considered in each separate dataset minus the one in the joint dataset, which in this case is, $d=K_{P2T}+K_{T2P}-K_{ALL}$. With 260.60 $\chi^{2}$ value and 12 degrees of freedom, a confidence level of over 99.99% was obtained. This indicates modeling P2T and T2P separately are more likely to present a superior fit compared to the joint dataset model.

Another likelihood ratio test was conducted to compare the differences between the random parameters model and their fixed parameters model using the test statistics below [31]:

(7) $${Χ}^{2}=-2\left[LL\left(\beta_{fixed}\right)-LL\left(\beta_{random}\right)\right]$$

where $LL\left(\beta_{fixed}\right)$ and $LL\left(\beta_{random}\right)$ are the log-likelihoods at the convergence of fixed parameters ordered probit model and random parameters ordered probit model estimated using the same dataset (All, P2T, and T2P), respectively. This ${Χ}^{2}$ statistic is a $\chi^{2}$ distribution with the degrees of freedom equal to the difference in the number of parameters between the two models. A list of $\chi^{2}$ value and the degrees of freedom for each dataset are presented in Table 3.

The test results were significant for these three crash datasets with the p-values all below 0.001, which gives more than 99.99% confident to believe that the random parameters ordered probit models outperforms the corresponding fixed ones.

## 6. Empirical Results and Discussion 

The random parameters ordered probit model was estimated through simulated maximum likelihood 200 Halton draws, which has been demonstrated to be an efficient method in producing accurate results for discrete choice models with low dimensionality of integration [37]. In addition, Halton draws provides a better simulation performance than random draws due to the dramatic speed gains with no degradation. The normal distribution was considered as the distribution for random parameters among other distributions including lognormal, triangular, and uniform distribution since previous studies have shown that normal distribution almost always outperforms other distributions [13,18,38].

Fixed and random parameters model estimation results for P2T and T2P crashes are presented in Table 4 and Table 5, respectively. The marginal effects of the random parameters model for these two datasets are shown in Table 6 and Table 7. The backward selection was performed to select the best subsets of the independent variables with a criterion of *p*-value >0.1. Hence, all estimated parameters included in the final model were statistically significant at a confidence level of 90% and the results were plausible as discussed below.

To start with, because of the difference in the potential characteristics of the collision order, the variables found statistically significant in the P2T model were significantly different from those found in the T2P model. It was also noteworthy that 6 of these variables determining injury severity gave generally similar results between P2T and T2P crashes. The details of each model were discussed separately.

### 6.1. P2T Crashes Variables

Regarding the model estimation results of P2T crashes, Table 4 shows that 16 variables have significant impacts on injury severity. A positive sign of a parameter indicates the probability of more severe outcomes (i.e., severe injury) increase, while less severe outcomes (i.e., no injury) decrease. In total, 2 out of 17 significant variables were determined to be random parameters and thus had a variable effect on injury severity outcome probabilities, i.e., male indicator and indicator for starting/stopped in the road before the crash.

#### 6.1.1. Person Characteristics 

Male occupant indicator and use of restraint system indicator tend to increase the probability of less severe injuries. Possible explanations are that males are physically stronger and have greater injury-sustaining capacity over females [39], and restraint system such as shoulder belt or lap belt tend to protect occupants from bumping into the wheel, dashboard or being ejected from the seat [40]. In addition, restraint systems are mostly developed based on the body structure of men, which might be another possible reason to explain the decreased likelihood of injury severity for a male with the restraint system. However, the male indicator resulted in a normally distributed random parameter with a mean of −0.2793 and a standard deviation of 0.5610, indicating a mixed impact on injury severity. In particular, 30.9% of males (above zero) in P2T crashes were more likely to experience the risk of injuries than females, probably due to the aggressive driving behavior of males, which is also consistent with previous studies as being identified by several researchers [41,42]. In addition, two age groups, age under 25 and age above 64, were found to have a strong association with injury severities in the P2T model. Occupants above 64 were more likely to experience more severe injuries while those under 25 tend to have a lower risk of sustaining more severe injuries. This finding is likely attributable to the lower bone mass density and longer reaction time for elders than youngsters [43,44].

#### 6.1.2. Vehicle Characteristics 

The model results also suggested that three vehicle characteristics were statistically significantly associated with injury severities. As consistent with past research [45], trucks in rear-end collisions with passenger cars have natural advantages because of their heavier weight and larger body and therefore are more likely to experience less severe injuries. Vehicles with one or more trailing units also decrease the likelihood of severe injuries (significant in P2T crashes only), which is possibly due to more proficient driving experience and training of drivers of these vehicles as compared to those of vehicles without a trailing unit [18]. In addition, in line with common sense, the results indicated a positive association between driver drinking in vehicle and injury severity for P2T crashes in this study. Specifically, drink and driving led to an increase in the likelihood of possible injury, evident injury, and severe injury by 0.0567, 0.0476, and 0.0139, respectively.

#### 6.1.3. Roadway and Environment 

Surface conditions and lighting conditions were found to affect severity outcomes. Interestingly, the model results showed that severe injury accidents happened less on the icy or snowy road surface than normal or dry road surface for P2T crashes only. It is likely a result of more cautious driving behavior on icy or snowy surfaces [13]. This finding was consistent with previous studies where normal road surface condition was found to provoke more severe accidents [45,46]. Whereas for lighting conditions, the dark and the dawn or dusk conditions were found to be significant in affecting injury outcomes, especially for dawn and dusk conditions (significant for P2T model only). The likelihood of severe injury raised by 0.0540, 0.0453, and 0.0132 for possible injury, evident injury, and severe injury, respectively. This result is perhaps due to the poor driving vision in dawn or night and gives passenger car drivers less time to perceive the forward environment and react accordingly [47].

#### 6.1.4. Crash Mechanism 

Four variables in crash mechanism showed a strong association with injury outcomes in P2T crashes. Going straight before crash increased the probability of more severe injury compared to the vehicle making curve or changing lanes. On the contrary, the two critical event indicators of other vehicles stopped in lane and other vehicles in lane traveling in the same direction while decelerating were found to be negatively associated with injury severity. Specifically, these two critical event indicators (significant for the P2T model only) implied the occupant sat in the striking vehicle. Past studies have shown that the front vehicle tended to suffer high levels of injury severity in a rear-end collision [5], which explains why the striking vehicle suffers less severe injury than the struck vehicle. Similar model estimation results were also found in ALL dataset. It showed that the collision order tended to determine injury severity levels. Specifically, when the passenger-car was struck by a truck as opposed to the other way around, the probability of severe injury increased (Please refer to Table A1 and Table A2 in Appendix A for complete model results for ALL dataset). The variable reflecting starting or stopped in the road before the crash was found to be normally distributed with a mean of −0.9347 and a standard deviation of 1.0185 in the P2T model only, implying its impact on injury severity varied across individuals. That is to say, 82.1% of individuals (below zero) who starting or stopped in the road before a crash in this study experienced less injury while the rest of 17.9% of observations (above zero) were more likely to sustain severe injury.

#### 6.1.5. Temporal Characteristics 

Two variables were found to have a positive impact on injury severity for P2T crashes only. In particular, the probability of severe injury slightly increased for the crashes occurring during weekdays and the spring. Similar results have been found in previous studies [22].

### 6.2. T2P Crashes Variables

Turning to the model estimation results for T2P crashes, as shown in Table 5, six variables were found to have similar impact on the injury severity as those in P2T crashes, i.e., male indicator, use of restraint system, truck indicator, dark condition, going straight before crash, and critical event for other vehicle stopped in lane. Moreover, driver indicator, age between 55 and 64, other vehicles in lane traveling in the same direction with higher speed, and summer indicators were showed to be statistically significant in determining injury severity for T2P crashes only. In addition, truck and other vehicles stopped in lane indicators were found to have a random effect on injury severity outcomes. This section will only discuss these six variables.

Regarding person type, the driver was found to decrease the possibility of severe injury in T2P crashes, likely due to the seat position and self-protect mechanism of drivers in a rear-end collision. Another variable in person characteristics reflecting the age between 55 and 64 showed a positive association with severe injury severity. This finding is in line with past studies as the physiological strength and injury-sustaining capability of this age group are relatively low [18].

As illustrated in the above section, it is evident that occupants in trucks experience a lower injury than in passenger cars in a rear-end collision. However, the effect seems to be mixed as the truck indicator was found to be normally distributed with a mean of −3.2639 and a standard deviation of 1.8687. It implied a 4.0% of observations (above zero) were more likely to obtain more severe injury while 96% of observations (below zero) were found to experience less injury in T2P crashes. This small proportion could refer to the aberrant driving behavior of truck drivers or unused safety measurement of truck occupants.

The indicator representing other vehicles stopped in the lane was also found to have a combined effect on injury severity, resulting in a normal distribution with a mean of −0.6351 and a standard deviation of 0.9271. This indicated that except for the 75.3% of observations who were more likely to be more severely injured (discussed before), 24.67% of occupants (above zero) were more likely to experience less severe injury. One possible explanation could be that these drivers in the rear vehicle tend to maintain a safer stopping distance, therefore, it gives the driver enough time to react to the sudden brakes of the front vehicle.

The model also indicated that other vehicles in lane traveling in the same direction with a higher speed slightly decreased the probability of more severe injury. A possible reason could be the minor speed difference between truck and passenger car, which reduced the injury severity accordingly.

In T2P crashes, only one temporal characteristic variable was found to be statistically significant. The indicator reflecting summer time slightly increased the severe injury probability. The high temperature in the summertime has been suggested as an important factor leading to an increase in stress and a decrease in motor skill performance for drivers [48].

Overall, separate injury severity models based on P2T crashes and T2P crashes can shed light on identifying the significant contributing factors. However, similar to previous research on accident severity, limitations also exist which should be taken into account before applying its findings. One limitation is the underreporting for minor property damage only and no significant personal injury in the GES database, and second, potentially crucial variables such as the avoidance maneuver taken by the driver within crash have been neglected due to the lack of available data. The findings would be more generalizable if the dataset were abundant to provide additional information about truck-involved rear-end collisions.

## 7. Conclusions

Injury severity of rear-end crash is of great concern to public health and environment. This study employed a random parameter ordered probit modeling framework to investigate the impact of contributing factors on injury severity of truck-involved rear-end collisions. Using the data from NASS-GES, separate models for the most severely injured occupant of truck-involved rear-end crashes (ALL model), passenger cars strike trucks (P2T model), and passenger cars stuck by trucks (T2P model) were developed. The likelihood ratio tests were conducted to evaluate the goodness of fit for these three models. The model estimation results demonstrated the necessity of modeling P2T and T2P crashes separately to analyze truck-involved rear-end collisions.

Similarities and differences were observed across the two models in terms of person characteristics, vehicle characteristics, roadway and environment, crash mechanism, and temporal characteristics. Some variables are significant only in P2T crashes, but not in T2P crashes, and vice versa. Key differences include age group, trailing units, drinking driving, road surface and critical events which made the crash imminent. For example, age under 25 and age above 64 was found to be significant in P2T crashes, while age between 55 and 64 indicator was only found significant in T2P crashes. Moreover, driver drinking in vehicle and dawn or dusk indicators were found to have a significant impact on increasing the injury levels. Furthermore, variables reflecting male, truck, starting or stopped in the road before crash, and other vehicles stopped in the lane were found to have a mixed impact on injury severity. In terms of these variables, a majority of observations showed an increase of probability in less severe injuries.

The results obtained from the developed models in this study have a number of practical implications. First, the use of the restraint system was found to decrease the probability of severe injury, suggesting that measurement in increasing seatbelt or lap belt compliance among truck and passenger-car occupants is needed. Second, age above 55 is more likely to experience injury severely, indicating a special carefulness targeting this age group is necessary to reduce injury severity. Third, it was found that severe injury increased under dark, dawn, or dusk conditions, suggesting that safety and enforcement agencies should seek extra instruments during dawn, dusk, and dark conditions. Fourth, a vehicle stopped in the lane before crash tends to decrease the probability of severe injury, therefore a kind reminder to keep a safe stopping distance between vehicles is necessary to allow enough reaction time for rear vehicles in a rear-end crash. Lastly, temporal variables reflecting weekdays, spring, and summer were found to be positively associated with severe injuries, indicating more attentions are needed by safety and enforcement agencies in these particular periods.

## Figures and Tables

**Table 1:** Injury severity studies of truck-involved crashes.

Authors	Truck Definition	Dependent Variable and Scale	Model	Key Findings
Duncan et al. (1998) [4]	Rigs carrying single tractor trailers	Injury severities of passenger car occupant with KABCO scales (1175 observations)	Ordered probit	Factors including dark condition, high-speed differentials, high-speed limits, grades, being in a car struck to the rear, drunk driving, and being female were found to be significant in contributing injury severities of truck-involved crashes.
Chang and Mannering (1999) [6]	A single unit or combination truck with GVWR exceeding 10,000 lb	Injury severities of most severely injured occupants with 3-level, property damage only, possible injury, and injury/fatality (17,473 vehicles)	Nested logit	Comparing to non-truck-involved accidents, factors including high-speed limits, crash occurring when making right or left turn, rear-end collision was found significant only for truck-involved crashes.
Khattak et al. (2003) [15]	Undefined	Injury severities of most severely injured occupant with KABCO scale (5163 crashes)	Ordered probit	Dangerous driving behavior, including speeding, alcohol/drug use, and non-use of restraints in single-vehicle truck crashes, significantly increased the injury severity of truck occupants.
Khorashadi et al. (2005) [10]	Trucks with GVWR over 10,000 lb	Injury severities of driver drawn randomly from crash vehicles with 4-level, no injury, complain of pain, visible injury, severe/fatal injury (17,372 vehicles)	Multinomial logit	Several factors, such as alcohol/drug use, were showed to have different influences on driver injury severity between rural and urban areas.
Lemp et al. (2011) [16]	Vehicles with GVWR over 10,000 lb	Two models: • Maximum injury severity suffered by any vehicle occupant with 4-level, no/possible injury, non-capacitating injury, capacitating injury, fatality (1894 observations) • Maximum injury severity suffered by any person involved in a crash with 3-level, non-capacitating injury, capacitating injury, fatality (922 observations)	Ordered probit and heteroskedastic ordered probit	The likelihood of fatalities and severe injury increased with the number of trailers but decreased with truck length and GVWR.
Chen and Chen (2011) [11]	Single-unit truck, tractor with a semi-trailer, and tractor without a semi-trailer	Injury severities of truck drivers with 3-level, no injury, possible/non-incapacitating injury, incapacitating injury/fatal (19,741 crashes)	Mixed logit/Random Parameters Logit	Sixteen variables were found to be only significant in single-vehicle crashes, whereas another sixteen factors were showed significance only in multi-vehicle crashes on a rural highway.
Zhu and Srinivasan (2011) [17]	Commercial vehicle weighing more than 10,000 lb	Injury severities of most severely injured occupants with 3-level, non-incapacitating injury, incapacitating injury, killed (953 crashes)	Ordered probit	Driver behavior variables, including driver distraction, alcohol use, and emotional factors, were found to have a statistically significant impact on severe injury.
Chang and Chien (2013) [12]	Vehicles with GVWR over 10,000 lb	Injury severities of the driver with 3-level, fatality, injury, and no-injury (1620 observations)	Classification and regression tree	Drunk-driving was the most detrimental factor for the injury severity of truck accidents.
Islam and Hernandez (2013) [18]	Tractor-trailer, single-unit truck, or cargo van with GVWR greater than 10,000 lb	Injury severities of most severely injured occupants with KABCO scales (8291 observations)	Random parameters ordered probit	The injury severity level was influenced by several complex interactions among factors related to human, vehicle, environment, and crash mechanism.
Islam et al. (2014) [19]	Undefined	Injury severities of most severely injured occupants with 3-level, major injury, minor injury, possible/no injury (8171 observations)	Mixed logit/Random Parameters Logit	There were differences in the influence on injury severity resulting from large truck at-fault accidents between rural and urban locations.
Pahukula el al. (2014) [14]	Undefined	The maximum level of injury sustained by the driver with 3-scale, severe injury, minor injury, no injury (11,560 observations)	Mixed logit/Random Parameters Logit	Traffic flow, light conditions, surface conditions, time of year, and percentage of trucks on the road were shown to have considerable differences in injury severity in different periods.
Naik et al. (2016) [13]	Single-vehicle trucks	Injury severities of a truck driver with 4-level, fatal/disabling injury, visible injury, possible injury, no injury/property damage only (1721 crashes)	Random parameters ordered logit and multinomial logit	Wind speed, rain, and warmer air temperature increased injury severities to single-vehicle truck crashes.
Uddin and Huynh (2017) [22]	Undefined	Injury severities of most severely injured occupants with 3-level, major injury, minor injury, possible/no injury (41,461 observations)	Mixed logit/Random Parameters Logit	Asphaltic concrete surfaces decreased the likelihood of major injuries for truck occupants during night time.
Uddin and Huynh (2018) [20]	Hazmat large trucks	Injury severities of most severely injured occupants with 3-level, major injury, minor injury, no injury (1173 observations)	Random parameters probit	Male occupants, truck drivers, crashes occurring in rural locations, dark-unlighted conditions, dark-lighted conditions, and weekdays were associated with increased probability of major injuries.
Behnood and Mannering (2019) [21]	Any medium or heavy truck, excluding buses and motor homes, with GVWR greater than 10,000 lb	Injury severities of most severely injured occupants with 3-level, no injury, minor injury, severe injury (large truck crashes in Los Angeles from 2010 to 2017, amount unclear)	Mixed logit/Random Parameters Logit	The effect of factors that determine injury severity varied significantly across time-of-day/time-period combinations.

**Table 2:** Descriptive statistics (All, *n* = 8506; P2T, *n* = 4866; T2P, *n* = 3640).

Variable Name	ALL		P2T		T2P	
	Mean *	S.D.	Mean *	S.D.	Mean *	S.D.
Person Characteristics						
Male	0.644	0.479	0.699	0.459	0.570	0.495
Driver	0.807	0.395	0.847	0.360	0.753	0.431
Use of restraint system	0.969	0.174	0.959	0.199	0.982	0.133
Age under 25	0.218	0.413	0.235	0.424	0.195	0.396
Age between 25–54	0.591	0.492	0.598	0.490	0.580	0.494
Age between 55–64	0.116	0.321	0.105	0.307	0.131	0.337
Age above 64	0.075	0.264	0.061	0.240	0.094	0.291
Vehicle Characteristics						
Truck	0.325	0.469	0.375	0.484	0.259	0.438
Vehicle with one or more trailing units	0.148	0.355	0.167	0.373	0.124	0.329
Driver drinking in vehicle	0.030	0.169	0.049	0.217	0.003	0.055
Speeding	0.142	0.349	0.202	0.402	0.060	0.238
Roadway and Environment						
Curve roadway alignment	0.051	0.219	0.046	0.210	0.056	0.231
Dry road surface	0.832	0.374	0.816	0.388	0.854	0.353
Wet road surface	0.136	0.343	0.143	0.351	0.126	0.332
Icy or snowy road surface	0.032	0.176	0.041	0.198	0.020	0.140
Daylight	0.780	0.414	0.736	0.441	0.840	0.367
Dark	0.194	0.396	0.238	0.426	0.135	0.342
Dawn or dusk	0.026	0.158	0.026	0.159	0.025	0.156
Crash Mechanism						
Collision order with passenger-car as leading vehicle	0.428	0.495	0.000	0.000	1.000	0.000
Going straight before crash	0.512	0.500	0.636	0.481	0.346	0.476
Decelerating/accelerating in road before crash	0.163	0.369	0.120	0.325	0.220	0.415
Starting/stopped in road before crash	0.259	0.438	0.181	0.385	0.362	0.481
Making a curve before crash	0.026	0.159	0.027	0.161	0.025	0.155
Changing lanes before crash	0.041	0.197	0.036	0.186	0.047	0.212
Critical event-in another vehicle’s lane	0.096	0.294	0.064	0.244	0.138	0.345
Critical event-other vehicle stopped in lane	0.202	0.402	0.261	0.439	0.125	0.330
Critical event-other vehicle in lane traveling in same direction with lower steady speed	0.115	0.319	0.173	0.378	0.038	0.192
Critical event-other vehicle in lane traveling in same direction while decelerating	0.136	0.343	0.171	0.377	0.088	0.284
Critical event-other vehicle in lane traveling in same direction with higher speed	0.421	0.494	0.313	0.464	0.565	0.496
Critical event-other vehicle encroaching into lane	0.030	0.171	0.018	0.135	0.046	0.209
Temporal Characteristics						
Peak hour (6:00–9:00, 16:00–19:00)	0.425	0.494	0.439	0.496	0.406	0.491
Weekdays	0.248	0.432	0.259	0.438	0.233	0.423
Spring	0.252	0.434	0.250	0.433	0.255	0.436
Summer	0.249	0.432	0.238	0.426	0.264	0.441
Fall	0.269	0.444	0.265	0.441	0.275	0.447
Winter	0.230	0.421	0.248	0.432	0.205	0.404

*: The mean value can be interpreted as the percentage. For example, a mean value of 0.644 of male means that 64.4% of the sample is male.

**Table 3:** Likelihood ratio tests between fixed and random parameters models.

Dataset	ALL	P2T	T2P
$\chi^{2}$ value	137.22	20.33	67.87
Degrees of freedom	4	2	2
*p* value	<0.001	<0.001	<0.001

**Table 4:** Model estimation results for car-strike-truck crashes (P2T).

Variable	Fixed Parameters Model		Random Parameters Model	
	Coefficient	*t*-stat	Coefficient	*t*-stat
Constant	0.8122 ***	7.31	0.9972 ***	8.51
Person Characteristics				
Male	−0.1750 ***	−4.09	−0.2793 ***	−6.33
• Standard deviation of parameter density function			0.5610 ***	19.62
Use of restraint system	−0.9796 ***	−11.49	−1.1135 ***	−12.34
Age under 25	−0.1603 ***	−3.49	−0.1780 ***	−3.76
Age above 64	0.2202 ***	2.91	0.2263 ***	2.92
Vehicle Characteristics				
Truck	−0.8980 ***	−11.29	−1.0162 ***	−12.04
Vehicle with one or more trailing units	−0.6942 ***	−7.02	−0.8488 ***	−7.76
Driver drinking in vehicle	0.3776 ***	4.55	0.3941 ***	4.77
Roadway and Environment				
Icy or snowy road surface	−0.2569 **	−2.34	−0.2676 **	−2.39
Dark	0.2127 ***	4.33	0.2387 ***	4.73
Dawn or dusk	0.3620 ***	3.26	0.3749 ***	3.19
Crash Mechanism				
Going straight before crash	0.1314 **	2.26	0.1230 **	2.06
Starting/stopped in road before crash	−0.2948 ***	−3.31	−0.9347 ***	−6.62
• Standard deviation of parameter density function			1.0185 ***	9.83
Critical event-other vehicle stopped in lane	−0.3000 ***	−5.75	−0.3161 ***	−5.93
Critical event-other vehicle in lane traveling in same direction while decelerating	−0.3348 ***	−5.75	−0.3581 ***	−6.03
Temporal Characteristics				
Weekdays	0.0863 **	1.97	0.1014 **	2.22
Spring	0.0790 *	1.78	0.0843 *	1.81
Thresholds				
Mu(01)	0.5657 ***	29.25	0.6230 ***	27.91
Mu(02)	1.3332 ***	39.52	1.4623 ***	37.97
Number of observations	4866		4866	
Restricted log likelihood	−4318.1191		−4318.1191	
Log likelihood function	−3747.5979		−3737.4327	
Akaike Information Criterion (AIC)	7533.2		7516.9	

Note: ***, **, * refer to Significance at 1%, 5%, 10% level.

**Table 5:** Model estimation results for truck-strike-car crashes (T2P).

Variable	Fixed Parameters Model		Random Parameters Model	
	Coefficient	*t*-stat	Coefficient	*t*-stat
Constant	0.7603 ***	4.89	0.7844 ***	5.33
Person Characteristics				
Male	−0.1550 ***	−3.69	−0.1458 ***	−3.45
Driver	−0.2093 ***	−4.65	−0.1979 ***	−4.27
Use of restraint system	−0.3445 **	−2.34	−0.3475 **	−2.55
Age between 55–64	0.1140 *	1.93	0.1242 **	1.97
Vehicle Characteristics				
Truck	−1.5714 ***	−17.64	−3.2639 ***	−14.86
• Standard deviation of parameter density function			1.8687 ***	12.81
Roadway and environment				
Dark	0.2389 ***	4.11	0.2762 ***	4.69
Crash mechanism				
Going straight before crash	0.1630 ***	3.20	0.1469 ***	2.75
Critical event-other vehicle stopped in lane	−0.2350 **	−2.22	−0.6351 ***	−3.86
• Standard deviation of parameter density function			0.9271 ***	6.64
Critical event-other vehicle in lane traveling in the same direction with higher speed	−0.1117 **	−2.23	−0.1206 **	−2.36
Temporal Characteristics				
Summer	0.1813 ***	4.04	0.1730 ***	3.72
Thresholds				
Mu (01)	1.0440 ***	41.65	1.0936 ***	38.90
Mu (02)	1.9092 ***	47.81	2.0019 ***	44.46
Number of observations	3640		3640	
Restricted log likelihood	−3934.3070		−3934.3070	
Log likelihood function	−3464.1798		−3430.2447	
Akaike Information Criterion (AIC)	6954.4		6890.5	

Note: ***, **, * refer to Significance at 1%, 5%, 10% level.

**Table 6:** Marginal effects for car-strike-truck crashes (P2T).

Variable	Marginal Effects			
	Severe Injury	Evident Injury	Possible Injury	No Injury
Person Characteristics				
Male	−0.0074	−0.0291	−0.0395	0.0761
Use of restraint system	−0.0855	−0.1737	−0.1313	0.3904
Age under 25	−0.0037	−0.0162	−0.0243	0.0441
Age above 64	0.0066	0.0249	0.0325	−0.0640
Vehicle Characteristics				
Truck	−0.0210	−0.0855	−0.1265	0.2330
Vehicle with one or more trailing units	−0.0113	−0.0560	−0.0974	0.1647
Driver drinking in vehicle	0.0139	0.0476	0.0567	−0.1182
Roadway and environment				
Icy or snowy road surface	−0.0046	−0.0218	−0.0350	0.0614
Dark	0.0064	0.0251	0.0339	−0.0654
Dawn or dusk	0.0132	0.0453	0.0540	−0.1125
Crash mechanism				
Going straight before crash	0.0027	0.0116	0.0170	−0.0314
Starting/stopped in road before crash	−0.0124	−0.0609	−0.1055	0.1787
Critical event-other vehicle stopped in lane	−0.0062	−0.0276	−0.0424	0.0762
Critical event-other vehicle in lane traveling in the same direction while decelerating	−0.0063	−0.0295	−0.0469	0.0827
Temporal Characteristics				
Weekdays	0.0025	0.0101	0.0143	−0.0269
Spring	0.0020	0.0084	0.0119	−0.0223

**Table 7:** Marginal effects for truck-strike-car crashes (T2P).

Variable	Marginal Effects			
	Severe Injury	Evident Injury	Possible Injury	No Injury
Person Characteristics				
Male	−0.0019	−0.0115	−0.0346	0.0479
Driver	−0.0028	−0.0166	−0.0471	0.0666
Use of restraint system	−0.0068	−0.0347	−0.0825	0.1240
Age between 55–64	0.0018	0.0104	0.0296	−0.0417
Vehicle Characteristics				
Truck	−0.0369	−0.1526	−0.3941	0.5836
Roadway and environment				
Dark	0.0045	0.0251	0.0659	−0.0955
Crash mechanism				
Going straight before crash	0.0020	0.0118	0.0349	−0.0487
Critical event-other vehicle stopped in lane	−0.0046	−0.0340	−0.1342	0.1728
Critical event-other vehicle in lane traveling in the same direction with higher speed	−0.0015	−0.0095	−0.0286	0.0396
Temporal Characteristics				
Summer	0.0024	0.0143	0.0412	−0.0579

## Appendix A

**Table A1:** Model estimation results for truck-involved rear-end crashes (ALL).

Variable	Fixed Parameters Model		Random Parameters Model	
	Coefficient	*t*-stat	Coefficient	*t*-stat
Constant	0.8045 ***	0.0859	1.1960 ***	0.0904
Person Characteristics				
Male	−0.1610 ***	0.0297	−0.2875 ***	0.0321
• Standard deviation of parameter density function			0.6950 ***	0.0226
Driver	−0.1048 ***	0.0348	−0.1444 ***	0.0371
Use of restraint system	−0.8249 ***	0.0729	−1.0177 ***	0.0742
Age under 25	0.1219 ***	0.0337	−0.1354 ***	0.0360
Vehicle Characteristics				
Truck	−1.1121 ***	0.0486	−1.4758 ***	0.0549
Vehicle with one or more trailing units	−0.4815 ***	0.0736	−0.6423 ***	0.0805
Driver drinking in vehicle	0.4337 ***	0.0771	0.5799 ***	0.0788
Roadway and Environment				
Icy or snowy road surface	−0.2256 ***	0.0869	−0.2395 **	0.0961
Dark	0.2258 ***	0.0374	0.2865 ***	0.0398
Dawn or dusk	0.2350 ***	0.0846	0.2659 ***	0.0891
Crash Mechanism				
Collision order with passenger-car as leading vehicle	0.2136 ***	0.0349	0.1708 ***	0.0379
Going straight before crash	0.1909 ***	0.0342	0.1108 ***	0.0371
• Standard deviation of parameter density function			0.7211 ***	0.0235
Critical event-other vehicle stopped in lane	−0.3177 ***	0.0413	−0.6448 ***	0.0493
• Standard deviation of parameter density function			0.8951 ***	0.0405
Critical event-other vehicle in lane traveling in same direction while decelerating	−0.3147 ***	0.0473	−0.6994 ***	0.0587
• Standard deviation of parameter density function			1.0258 ***	0.0517
Temporal Characteristics				
Summer	0.1034 ***	0.0318	0.1262 ***	0.0344
Mu(01)	0.7886 ***	0.0157	1.0179 ***	0.0211
Mu(02)	1.5887 ***	0.0256	2.0633 ***	0.0343
Number of observations	8506		8506	
Restricted log likelihood	−8459.0357		−8459.0357	
Log likelihood function	−7366.5881		−7297.9798	
Akaike Information Criterion (AIC)	14,769.2		14,640.0	

Note: ***, **, * refer to Significance at 1%, 5%, 10% level.

**Table A2:** Marginal effects for truck-involved rear-end crashes (ALL).

Variable	Marginal Effects			
	Severe Injury	Evident Injury	Possible Injury	No Injury
Person Characteristics				
Male	−0.0030	−0.0259	−0.0640	0.0929
Driver	−0.0015	−0.0130	−0.0323	0.0468
Use of restraint system	−0.0360	−0.1585	−0.1867	0.3812
Age under 25	−0.0011	−0.0107	−0.0297	0.0415
Vehicle Characteristics				
Truck	−0.0115	−0.0951	−0.2648	0.3714
Vehicle with one or more trailing units	−0.0035	−0.0380	−0.1267	0.1682
Driver drinking in vehicle	0.0115	0.0725	0.1258	−0.2098
Roadway and Environment				
Icy or snowy road surface	−0.0016	−0.0168	−0.0509	0.0693
Dark	0.0034	0.0277	0.0642	−0.0952
Dawn or dusk	0.0034	0.0271	0.0598	−0.0904
Crash Mechanism				
Collision order with passenger-car as leading vehicle	0.0016	0.0147	0.0379	−0.0542
Going straight before crash	0.0010	0.0093	0.0245	−0.0348
Critical event-other vehicle stopped in lane	−0.0038	−0.0405	−0.1296	0.1739
Critical event-other vehicle in lane traveling in the same direction while decelerating	−0.0036	−0.0396	−0.1353	0.1784
Temporal Characteristics				
Summer	0.0013	0.0112	0.0282	−0.0406
